# β-Glucan Synthesis Gene Family in Crops: Advances in Classification, Function, and Regulation

**DOI:** 10.3390/cimb47120983

**Published:** 2025-11-25

**Authors:** Xin Huang, Xuan Wu, Yaodan Zhang, Jiayi Jin, Cuomu Mingma, Yang Bai, Hui Zhao, Yajie Liu, Guowu Yu

**Affiliations:** 1 National Demonstration Center for Experimental Crop Science Education, Sichuan Agricultural University, Chengdu 611130, China; 202200322@stu.sicau.edu.cn (X.H.); 202200312@stu.sicau.edu.cn (X.W.); zhangyaodan@stu.sicau.edu.cn (Y.Z.); 202201472@stu.sicau.edu.cn (J.J.); zhaohui@sicau.edu.cn (H.Z.); 14048@sicau.edu.cn (Y.L.); 2 State Key Laboratory of Crop Gene Exploration and Utilization in Southwest China, College of Agronomy, Sichuan Agricultural University, Chengdu 611130, China

**Keywords:** Csl subfamilies, (1,3;1,4)-β-glucan, gene regulation network, cereal crops, MLGs

## Abstract

β-glucan is an important component of crop cell walls, and its chemical structure is closely related to its physiological function. Among them, (1,3;1,4)-β-glucan (mixed-linkage β-glucan, MLGs) is a unique type of β-glucan in graminaceous crops. In recent years, the identification and functional analysis of the β-glucan synthesis gene family have provided key molecular targets for quality improvement of crops. This review systematically examines the classification, structure, and function of the β-glucan synthetic gene family, focusing on the regulatory network. This will lay a theoretical foundation for the further improvement of the β-glucan content in crops.

## 1. Introduction

β-glucan functions widely in medicine, the food industry and crop breeding [1]. In the medicine and healthcare industry, (1,3,6)-β-glucan acts as an immunomodulator via intestinal receptors [2,3]; paramylon-represented (1,3)-β-glucan has anti-obesity, anti-diabetic and immune-stimulating effects [4,5]; cellulose-represented (1,4)-β-glucan aids weight loss by increasing satiety [6]; and graminaceous (1,3;1,4)-β-glucan (MLG) delays gastric emptying, reduces postprandial blood glucose, and promotes the production of short-chain fatty acids [7,8]. Moreover, daily intake of 3 g barley β-glucan reduces LDL-C levels, while its gelatin sponges alleviate immunosuppression [7] and improve wound healing [9]. In food processing, MLG serves as a natural thickener, e.g., 1% oat β-glucan increases yogurt viscosity by 30% and improves taste [10]. Fungal β-glucan promotes the growth of lactic acid bacteria by shortening hysteresis and increasing the growth rate [11]. In crop breeding, callose, a (1,3)-β-glucan with β-1,6 branches, is vital for plant development and stress response, so regulating its content helps breed excellent varieties [12]. On the other hand, β-glucan nanoparticles can also promote plant growth and control tomato Fusarium wilt [13].

**Figure 1:**
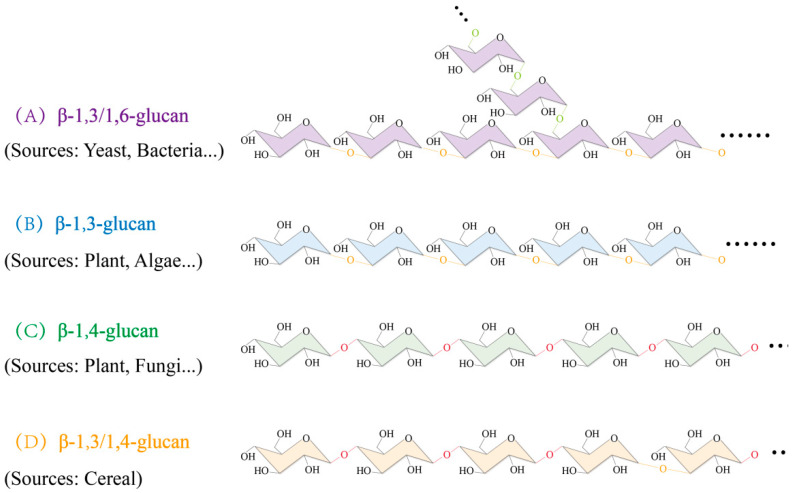
Molecular structures of different branching types of β-glucan and their main sources: green glycosidic bond for β-1,6 glycosidic bond, orange glycosidic bond for β-1,3 glycosidic bond, and red glycosidic bond for β-1,4 glycosidic bond.

The diverse functions of β-glucan are due to its structure. β-glucans are polysaccharide molecules formed by the β-glycosidic linkages of UDP-Glc, and several types exist. β-1,3-glucan is a long chain of UDP-Glc linked by (1,3)-β-glucan linkages (Figure 1B). The UDP-Glc units that make up (1,3)-β-glucan usually branch at the sixth carbon and form side chains through β-1,6 glycosidic bonds to form (1,3;1,6)-β-glucan (A in Figure 1) [14]. (1,4)-β-glucan is a long chain of UDP-Glc linked by β-1,4 glycosidic linkages (Figure 1C), and fibrillar cellulose is a long chain of (1,4)-β-glucans [6]. Among the various types, MLG is a linear polysaccharide formed by a mixture of β-1,3 and β-1,4 glycosidic linkages linking UDP-Glc units (Figure 1D). The linear structure of MLG consists of repeating oligosaccharide units, typically 2–3 glucose residues linked by β-1,4 glycosidic bonds and forming cellotriose or cellobiose segments, which are then connected to the next oligosaccharide unit via a single β-1,3 glycosidic bond (e.g., cellotriose-β-1,3-cellobiose). In this structure, the number of β-1,4 glycosidic bonds is approximately 2–3 times that of β-1,3 glycosidic bonds, and the arrangement of the glycosidic bonds is somewhat regular rather than completely random, a feature closely related to its water solubility and physiological functions [15,16].

β-glucans are widely found in plants, algae, fungi and bacteria, but the distribution of different types of β-glucans varies among organisms [16]. (1,3)-β-glucan is abundant in fungi, plants, and algae, but mainly in algae [17]. (1,3;1,6)-β-glucan is mainly found in yeast and bacteria, and it is especially abundant in mushrooms [2,18]. (1,4)-β-glucan is mainly found in plants and is a key structural component of plant cell walls [19]. MLG is found only in graminaceous crops [20], and its distribution varies among crops. For example, oats contain 6–8% (*w*/*w*) β-glucan and barley contains 4–10% (*w*/*w*) β-glucan [15]. Moreover, barley and oats have more MLG than other cereals, which better meet human dietary and industrial requirements [1].

In crops, the distribution of β-glucan is cell- and tissue-specific [21]. For example, in oat starchy endosperm cells, MLG is a major component of the cell wall, forming a network structure with cellulose and arabinoxylan to support cell morphology; in wheat aleurone layer cells, the cell wall matrix is dominated by cross-linked structures of (1,4)-β-glucan and arabinoxylan, where β-glucan exhibits extremely low water solubility due to high cross-linking. As a layer of living cells in the outer endosperm, the special structure of the aleurone layer cell wall is closely related to its functions of nutrient storage and response to germination signals [22]. Callose, a (1,3)-β-glucan, is mostly deposited in the cell wall near the neck region of plasmodesmata [23].

This review focuses on the classification, function, and regulation of β-glucan synthesis gene families (*Ces*/*CalS*/*Csl*), with an emphasis on MLG, to provide a theoretical basis for crop quality improvement.

## 2. Classification of β-Glucan Synthesis Gene Families

β-glucan synthesis relies on three major gene superfamilies: cellulose synthases (*Ces*), callose synthases (*CalS*), and cellulose synthase-like (*Csl*). These three superfamilies exhibit significant species-specific differentiation across different crops, and their family size, subfamily composition, and functional specialization are closely associated with crop evolution and the demand for cell wall components.

### 2.1. Core Gene Families Involved in β-Glucan Synthesis and Their Functional Features

#### 2.1.1. Core Functional Localization of the Three Gene Families

The core members of the Ces family are cellulose synthase A (CesA), which belong to the GT2D family in the CAZy database. They are responsible for catalyzing the directional polymerization of (1,4)-β-glucan (cellulose), serving as the fundamental structural support for plant cell walls [24,25,26]. Their function is highly conserved and indispensable in all land plants, and they need to form multi-subunit complexes (e.g., the primary wall complex composed of CesA1/CesA3/CesA6 in Arabidopsis) to exert their roles [27].

The *CalS* family belongs to the *GT48* family and specifically catalyzes the synthesis of (1,3)-β-glucan (callose), requiring the formation of complexes with auxiliary proteins such as sucrose synthase (SuSy) and Rop1 [28]. Although callose has a low content in plants, it regulates pollen tube development, plasmodesmata permeability, and stress responses. The size of this family varies with the adaptive needs of different species [29,30,31].

The *Csl* family is a member of the CAZy *GT2D* family and represents the “core of functional differentiation” in β-glucan synthesis, responsible for the synthesis of hemicellulose, pectin, and the gramineous plant-specific (1,3;1,4)-β-glucan (MLG) [32]. The distribution of its subfamilies (A–J) is strongly associated with crop types. For example, *CslF*, *CslH*, and *CslJ* exist only in monocot crops, while dicots are characterized by *CslM* (which undergoes convergent evolution with *CslJ*) [33], reflecting the adaptive evolution of gene families to the diversity of cell wall components.

#### 2.1.2. Distribution and Functional Characteristics of Csl Gene Subfamilies

The subfamilies of cellulose synthase-like proteins (Csl-like proteins) have different distributions in organisms. *CslA* and *CslB* are present in a variety of plants and microorganisms; *CslC* and *CslD* are present in monocotyledons and dicotyledons, but genes with similar functions may be present in microorganisms and algae; *CslF* and *CslH* are present in monocotyledons and a variety of algae and microorganisms; and *CslJ* is widespread in plants and microorganisms, and some members of its lineage can direct β-glucan biosynthesis. In recent years, however, a newly identified gene lineage in dicotyledonous plants, CslM, has formed a reciprocally monophyletic eudicot–monocot clade with *CslJ*. The *CslM* lineage is widely distributed in dicotyledonous plants, while the *CslJ* branch, previously thought to be limited to the Poaceae family, is widely distributed in monocotyledonous plants [33,34,35,36,37].

The functions of the various subfamilies of cellulose synthase-like proteins (Csl-like proteins) also vary. Of the subfamilies J and A to H, the functions of some subfamily members (CslE/G/M/B) remain largely unknown, and the functions of the known subfamilies are shown in Table 1.

In crops, the expression of *Csl* gene family members exhibits spatio-temporal specificity, tissue specificity and complementarity in the expression of *Csl* gene family members. In the starchy endosperm development of barley, *HvCslF6* and *HvCslF9* transcripts predominate, and they share two transcriptional peaks: one occurs after endosperm cellularization, 4 to 8 days after pollination, while the second occurs at a later stage of grain development, 20–25 days after pollination [51,52]. Meanwhile, in barley, *HvCslF6* is highly expressed in the endosperm and dominates seed β-glucan synthesis, and *HvCslF9* is specifically expressed in the leaves in response to pathogen infection [51]. In common oats, members of the *CslF* subfamily are significantly overexpressed in stems; meanwhile, 30 *Csl* genes are upregulated in immature seeds [50]. In *Nelumbo nucifera*, different *Csl* subfamilies play distinct roles in organ development. Furthermore, under salt stress, the expression of *CslH* and *CslF* is synergistically upregulated to enhance cell wall mechanical strength [53]. Song analyzed the expression of *CesA*/*Csl* genes in different tomato tissues at various developmental stages and found that three *CslD* genes are specifically expressed in flowers, while five putative *Csl* genes are preferentially expressed in fruits [54]. Sipahi used RT-qPCR to detect the expression of 10 *CsCSL* genes in the flowers, leaves, roots, and stems of Cannabis sativa, revealing that these *CSL* genes exhibit differential expression across organs [55]. The β-glucan content of the double mutant (*CslF6*/*CslF9*) is significantly lower than that of the single mutants, suggesting that these genes function in a partially redundant yet synergistic manner [51]. Garcia-Gimenez knocked out the *CslF6* and *CslH1* genes in barley using CRISPR/Cas9 and found that the double-knockout mutants exhibit severe phenotypic defects, indicating functional complementation between these two genes [56].

### 2.2. Cross-Species Differences in β-Glucan Synthesis Gene Families

#### 2.2.1. Differences in Family Size and Subfamily Distribution Among the Three Families

Monocot plants are exemplified by rice, maize, barley, and oats. Due to the need to synthesize MLG (a gramineous-plant-specific cell wall component), the *Csl* family exhibits the characteristics of “expansion of specific subfamilies + functional specialization”: *CslF*, *CslH*, and *CslJ* are core subfamilies. Among them, the 2H chromosome of barley contains a 118 kb *CslF* gene cluster (including *HvCslF3/4/6/7/8/9/10*) [51]; the oat *Csl* family has 76 members, covering seven subfamilies (*CslA/C/D/E/F/H/J*) [50]; and the *CslC* subfamily in maize has expanded from 6 members in rice to 12, while the *CslH* subfamily has contracted from three to one, reflecting the differentiated demands for cell wall polysaccharide synthesis among different monocot crops [57]. The size of the *CalS* family is relatively conserved: barley contains 7 *CalS* genes [29,30,31], and rice contains approximately 10 (*OsCalS5* regulates anther callose deposition [58]), which is significantly fewer than the 32 CalS genes in the dicot rapeseed [29,30,31]. This is presumably related to the relatively stable demand for callose in the reproductive development of monocots.

Dicot plants are exemplified by Arabidopsis, cotton, tomato, and alfalfa. They do not need to synthesize MLG, thus lacking the *CslF, CslH*, and *CslJ* subfamilies, but have evolved a functionally similar *CslM* subfamily (forming a parallel monophyletic group with monocot *CslJ*, undergoing convergent evolution [33]). The overall size of the *Csl* family in dicots is smaller than that in monocots. For example, the Arabidopsis *Csl* family contains 9 subfamilies [32]; tomato contains 38 *CesA/Csl* members [54]; alfalfa has 37 CesA/Csl members [59]; while cotton, due to its polyploidy (allotetraploid), has 228 *Ces*/*Csl* family members divided into 11 subfamilies [60], reflecting the driving effect of polyploidization on gene family expansion. The size of the *CalS* family varies greatly. Arabidopsis contains 12 *CalS* genes (*AtCalS5* regulates pollen tube development [28]), and rapeseed contains 32 [29,30,31], presumably related to the demand for callose synthesis in dicots in response to complex stresses (e.g., pathogen infection).

To intuitively present the cross-species differences, the gene families related to β-glucan synthesis in major crops are compared by integrating data from the original text and reported genomic characteristics (Table 2).

#### 2.2.2. Evolutionary Patterns of the Csl Family Based on Phylogenetic Analysis

**Figure 2:**
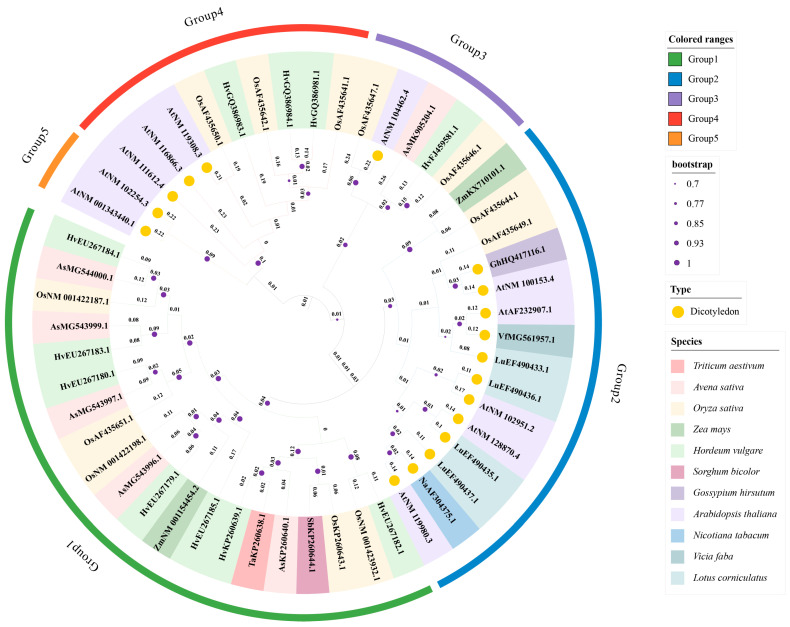
Phylogenetic tree illustrating the evolutionary relationships among 51 members of the Csl superfamily. The phylogenetic tree illustrates the evolutionary relationships among 51 members of the Csl superfamily. Evolutionary history was inferred using the neighbor-joining method, with the evolutionary distances calculated via the p-distance method. The analysis encompassed 51 encoded nucleotide sequences, including sites from the first, second, third, and non-coding regions. This phylogenetic analysis was conducted using MEGA12. The values on the branches in the diagram represent branch lengths, and the purple dots represent bootstrap values. All the members are divided into five groups according to the bootstrap values.

The Csl gene family is involved in the synthesis of polysaccharides (e.g., MLG), but its specific role in β-glucan synthesis remains unclear. The 51 classified and sequenced Csl gene family members from *A. thaliana* and various crops are divided into subfamilies A, C, D, E, F, H, and J based on their evolutionary relationships (Figure 2).

The phylogenetic tree divides the members into five groups, with *Csl* members from monocots and dicots forming independent clades. Group 1 includes *HvCslA, HvCslC, HvCslD, HvCslE, HvCslF, HvCslG, AsCslD, AsCslF, OsCslD, OsCslF, ZmCslD, TaCslF*, and *SbCslF*; Group 2 includes *AtCslD, NaCslD, LuCslD, VfCslD, GhCslD, OsCslE, OsCslG*, and *ZmCslF*; Group 3 includes *AtCslE, OsCslE, HvCslF*, and *AsCslF*; Group 4 includes *AtCslC, OsCslC, OsCslF*, and *HvCslF*; and Group 5 includes *AtCslA*.

Group 1 consists entirely of monocot members, and these members form a tight clade, reflecting the conservation of their function in MLG synthesis. Dicot members are mainly concentrated in Groups 2 and 5, e.g., Arabidopsis *AtCslD3 (AtNM104462.4)* and *AtCslG3 (AtNM128870.4)*. Only a few clades (e.g., monocot *OsCslD1 (OsAF435651.1)* and dicot *VfCslD1 (VfMG561957.1)* in Group 2) are intertwined, suggesting the existence of a common ancestor during early evolution. Different *Csl* subfamilies exhibit specific clustering in the phylogenetic tree, which correlates with their functions. The *CslF* subfamily (responsible for MLG synthesis) is almost entirely in Group 1; in addition to the aforementioned monocot members, it also includes rice *OsCslF4 (OsAF435647.1)* and *OsCslF12 (OsAF435650.1)*. The *CslD* subfamily (involved in cellulose synthesis) spans Groups 2–4, such as oat *AsCslD (AsMG543996.1, AsMG543999.1)* and *Arabidopsis AtCslD3 (AtNM104462.4)*. Its wide distribution reflects the conserved function of this ancient subfamily. The *CslA/C/G* subfamilies (synthesizing mannan/xyloglucan) exhibit moderate clustering; for example, barley *HvCslA9 (HvEU267185.1)* and *Arabidopsis AtCslA3 (AtNM100153.4)* form a small clade in Group 4, while barley *HvCslG1 (HvEU267180.1)* and *cotton GhCslG2 (GhHQ417116.1)* cluster in Group 2.

Members with long branches (e.g., barley *HvCslF10 (HvEU267184.1)* and rice *OsCslE1 (OsAF435649.1)*) have bootstrap values exceeding 0.9 and high genetic variation; they may have acquired new functions (e.g., responding to biotic stress) through amino acid substitutions in the “switch motif”. Members with short branches (e.g., Arabidopsis *AtCslA3 (AtNM100153.4)* and rice *OsCslD1 (OsAF435651.1)*) exhibit slow evolution, corresponding to conserved functions such as embryonic mannan synthesis and basic cell wall construction.

## 3. Progress in the Study of the Function of the β-Glucan Synthesis Gene Family

After clarifying the classification of the β-glucan synthesis gene family (*Ces/Csl/CalS*), its functional implementation mechanism is at the core of elucidating β-glucan synthesis—specifically, how members of different families collaborate to complete synthesis and how their structures support functions, which needs to be explored from three aspects: synthesis process, structural characteristics, and functional characterization.

### 3.1. Mechanism of β-Glucan Synthesis

#### 3.1.1. Substrate Synthesis, Transport, and Catalytic Polymerization

The synthesis of (1,3;1,6)-β-glucan relies on specific glucan synthases, such as the Fks protein in yeast [18]. In plants, although no typical (1,3;1,6)-β-glucan synthesis genes have been identified, exogenous application of (1,3;1,6)-β-glucan can enhance crop resistance to pathogenic bacteria [79]. (1,4)-β-glucan chains are directionally polymerized from UDP-Glc by the cellulose synthase (CesA) complex via transmembrane catalytic channels [80]. In crops, (1,3)-β-glucan is formed when CalS catalyzes the polymerization of UDP-Glc into glucan chains. Csl proteins also catalyze the polymerization of UDP-Glc to form short (1,3)-β-glucan chains, a process involved in MLG synthesis that will be emphasized below.

Glycosyltransferases, which catalyze MLG synthesis, are mainly localized in the plasma membrane and Golgi apparatus [81]. Crops produce glucose in leaves via photosynthesis; this glucose is then converted into glucose-6-phosphate in the cytoplasm by hexokinase. Glucose-6-phosphate is further converted into uridine diphosphate glucose (UDP-Glc), which is the direct donor for β-glucan synthesis. UDP-Glc is transported into the synthesis complex on the inner side of the plasma membrane via membrane transporters, providing substrates for subsequent polymerization reactions.

**Figure 3:**
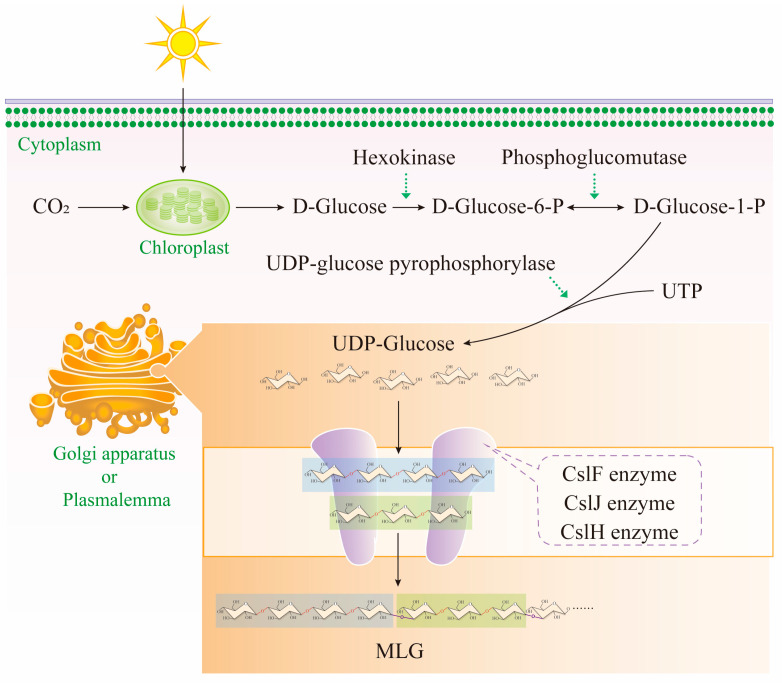
(1,3;1,4)-β-glucan synthesis pathway: black solid arrows indicate the direction of the substance reaction; green dashed arrows indicate enzyme catalysis.; the red glycosidic bonds are β-1,3 glycosidic bonds, and the purple glycosidic bonds are β-1,4 glycosidic bonds.

Csl proteins, as core catalytic enzymes, are anchored in the plasma membrane to form multimeric complexes. Their catalytic domains bind UDP-Glc via the conserved “Thr-Glu-Asp” (TED) motif and gradually extend glucan chains through β-1,4 glycosidic bonds [72,82]. Studies have shown that *CslF6* contains a “switch motif” at the entrance to its transmembrane channel, which dynamically adjusts the type of glycosidic bond by monitoring the spatial orientation of the second or third glucose residue in the nascent chain [72]. When specific amino acids in this motif (e.g., tryptophan) form hydrogen bonds with the substrate, *CslF6* preferentially catalyzes the formation of β-1,3 bonds, thereby introducing a mixed-linkage structure [72]. This structural regulatory mechanism is highly conserved in crops such as barley and wheat. The catalytic domain of CslF6 shares over 70% sequence similarity with cellulose synthase (*CesA*) but has acquired new functions through the substitution of residues specific to β-1,3 bond formation [83]. When the glucan chain reaches 200–500 glucose residues, *CslF6* terminates synthesis via an allosteric effect [84] (Figure 3). The synthesized MLG is transported outside the plasma membrane via secretory vesicles and cross-linked with other cell wall components (e.g., cellulose and arabinoxylan) to form the reticular structure of the cell wall [85].

#### 3.1.2. Subcellular Localization of β-Glucan Synthesis

The realization of the aforementioned catalytic polymerization process depends on the localization of synthetic proteins in specific subcellular structures—for only when they are localized to the correct membrane systems (Golgi apparatus/plasma membrane) can synthases access substrates and complete product transport. Therefore, clarifying the subcellular localization of MLG synthesis is critical for understanding its mechanism.

The more general view is that cellulose and callose are synthesized at the plasma membrane [86,87], and pectin and the rest of the polysaccharides that make up the cell wall are synthesized at the Golgi [88]. Pectin and hemicellulose are synthesized and assembled in the Golgi, secreted into the extracellular bodies, and eventually, modified and integrated into the cell wall [86]. Cellulose is synthesized by 40–60 nm diameter CesA protein complexes [86], which are assembled in the Golgi and transported to the plasma membrane where cellulose is synthesized [89]. To further verify the interaction between cytoskeletal components (AFs and MTs) and the enzymes studied (Sus, CalS, and CesA), Giampiero Cai separated the natural protein complexes of tobacco pollen tube plasma membrane by natural blue PAGE. CalS appears in a protein complex. The CalS complexes are then assembled in the endoplasmic reticulum and transported to the plasma membrane via Golgi bodies or vesicles that move along actin filaments [90], where they synthesize the callose [91]. The callose synthase complex in plants is centered on CalS. It is associated with sucrose synthase, UDP-glucose transfer, and multiple subunits, such as Rho-like protein, Phragmoplastin, and Annexin, forming a functional complex to act as a catalyst. SuSy and UGT are combined on the large hydrophilic ring of CalS. SuSy degrades Sucrose into UDP-glucose [28,92,93]. UDP-glucose is then transferred to the catalytic site of CalS by UGT as the substrate of the reaction [28]. UGT can bind to Rop 1 and interact with Phr. Rop 1 may control CalS activity through UGT [92]. Phr interacts with UGT in the plate-specific callose synthase complex. Phr can activate CalS. But the Phr protein may not be part of all callose synthase complexes [93]. ANN has GTPase activity and may be involved in Ca^2+^-mediated callose synthesis to cellulose synthesis [93].

However, there is controversy regarding the subcellular localization of MLG-synthesizing proteins, with the core debate centered on “dynamic Golgi apparatus-plasma membrane transport”.

First, there is the Golgi apparatus synthesis perspective. Through in vitro synthesis experiments on maize coleoptiles, Gibeaut were the first to confirm that MLG with a molecular weight exceeding 250 kD could be detected in Golgi apparatus membranes [28]. But in vitro synthesis experiments cannot simulate the “multi-organelle coordination” environment in plant cells and thus may overestimate the independent synthetic capacity of the Golgi apparatus. Kim further observed that barley *CslF6* is localized to the Golgi apparatus and that β-glucan is transported to the extracellular space via secretory vesicles—supporting the notion that the Golgi apparatus is the initial site of MLG synthesis [77]. However, such studies rely on in vitro membrane isolation technology, which not only may lead to organelle contamination but also involves differences between the in vitro synthesis environment (e.g., substrate concentration, pH) and the in vivo physiological state, making it difficult to fully reflect the actual synthesis process.

Second, there is also the plasma membrane assembly perspective. Wilson did not detect MLG-specific antibody signals in the Golgi apparatus of gramineous plants (e.g., wheat and barley); instead, they observed strong labeling on the plasma membrane. They hypothesized that after the Golgi apparatus synthesizes MLG precursors, the final assembly of MLG may be completed at the plasma membrane [94]. However, in vivo immunolocalization is limited by microscopic resolution and cannot distinguish between “Golgi-synthesized precursors” and “plasma membrane-assembled mature MLG”. Although Kim detected *CslF6* in the Golgi apparatus of Brachypodium distachyon, they did not detect MLG accumulation—this further supports the notion that “the plasma membrane is the key site for assembly” [95]. But subcellular fractionation technology cannot fully avoid cross-organelle contamination, which may lead to misjudgment of the MLG origin.

Currently, there are several key unresolved issues. If MLG synthesis requires the “Golgi synthesis—plasma membrane assembly” process, the vesicle-anchoring proteins (e.g., the Rab family) that mediate precursor transport have not yet been identified; additionally, it remains unknown whether there are species-specific differences in the MLG synthesis sites among different crops, and whether this is related to differences in the transmembrane structure of Csl proteins (e.g., the number of transmembrane helices [TMH]).

### 3.2. Structural Properties of β-Glucan Synthase

The catalytic polymerization process of MLG (e.g., the precise formation of β-1,3/β-1,4 glycosidic bonds) is not random but is determined by the specific structure of β-glucan synthases. Below is an elaboration on the core domains of β-glucan synthases and their association with catalytic function.

#### 3.2.1. Comparison of Protein Structures Between the CesA and Csl Families

CesA and CSL proteins both belong to the GT2D family and share a core catalytic domain; however, due to differences in specific motifs, they mediate the synthesis of cellulose and MLG, respectively, with a corresponding relationship between their structures and functions.

The core catalytic module on the cytoplasmic side is a functional unit shared by both types of proteins, responsible for UDP-glucose (substrate) binding and glycosidic bond formation. The D-motif cluster (D/DD/D×D) consists of 1–3 aspartic acid (D) residues, which stabilize the phosphate group of UDP-glucose by chelating Mg^2+^, thereby reducing the activation energy of the catalytic reaction [96,97]. The “D-D-D” triad motif in CesA and the “D×D” motif in *CslF* are functionally conserved and both serve as catalytic cores. The Q××RW motif, composed of glutamine (Q), arginine (R), and tryptophan (W), recognizes the glucose residue of UDP-Glc via hydrogen bonding and directionally regulates the formation of glycosidic bonds. In CesA, this motif specifically catalyzes the formation of β-1,4 bonds [98], whereas in *CslF*, it cooperates with the “switch motif” to switch between β-1,3 and β-1,4 bond formation [47,72]. Regarding the hydrophilic catalytic domain (PF00535), CesA and *CSL* share a common UDP-Glc binding pocket that directly encloses the substrate molecule, providing a microenvironment for the catalytic reaction [97]. Among these, the PF00535 domain of *CslF* shares more than 70% sequence homology with that of CesA; however, it acquires the ability to bind β-1,3 bond precursors through key amino acid substitutions (e.g., tryptophan replacing alanine) [51].

The transmembrane transport module in the membrane-embedded region mediates synthase anchoring and glucan chain export. Although there are quantitative differences between the two types of proteins, their functional logic is consistent. First, regarding transmembrane helices (TMHs): CesA contains 6–8 TMHs, which form a closed transmembrane channel that guides the directional export of (1,4)-β-glucan chains to the extracellular space [96]. *CslF* (e.g., barley *CslF6*) contains eight TMHs, forming a channel with a central pore size of approximately 1.2 nm. The pore size is directly related to the insertion frequency of β-1,3 bonds in MLG—a narrow pore size restricts the folding of mixed-linkage chains, leading to shortened MLG chain lengths [47,72]. Second, regarding membrane localization signals: the N-terminal and C-terminal sequences of TMHs determine the subcellular localization of synthases. The TMH terminal sequence of CesA guides its localization to the plasma membrane [89], whereas the TMH of *CslF* contains a Golgi-targeting signal, enabling it to first synthesize MLG precursors in the Golgi apparatus before transporting them to the plasma membrane via vesicles [78,95].

In the auxiliary regulatory module at the “cytoplasmic side–membrane interface,” the two types of proteins mediate complex assembly and activity regulation through different elements, reflecting functional specificity.

CesA has the zinc finger structure (zf-UDP), which directly binds UDP-glucose and supplements the substrate supply for the core catalytic module [99]. The RING domain does not directly participate in catalysis but regulates the assembly stability of the CesA trimer (CesA1/CesA3/CesA6) by mediating interactions between CesA and regulatory factors (e.g., kinase) [100].

*CslF* uniquely contains a “switch motif” located at the entrance to the TMHs, which includes a conserved tryptophan residue. By monitoring the spatial orientation of the second–third glucose residues in the nascent MLG chain, it dynamically switches the catalytic preference of the Q××RW motif. When tryptophan forms a hydrogen bond with the substrate, β-1,3 bond formation is prioritized; otherwise, β-1,4 bond formation is catalyzed. This directly determines the proportion of mixed linkages in MLG [47,72]. The auxiliary protein binding site: the loop region between the TMHs of *CslF* can bind Rop GTPases, enhancing the anchoring efficiency of the synthase on the Golgi membrane and ensuring the continuous synthesis of MLG precursors [47].

#### 3.2.2. Structural Differentiation and Functional Division of Labor Among Csl Family Members

**Figure 4:**
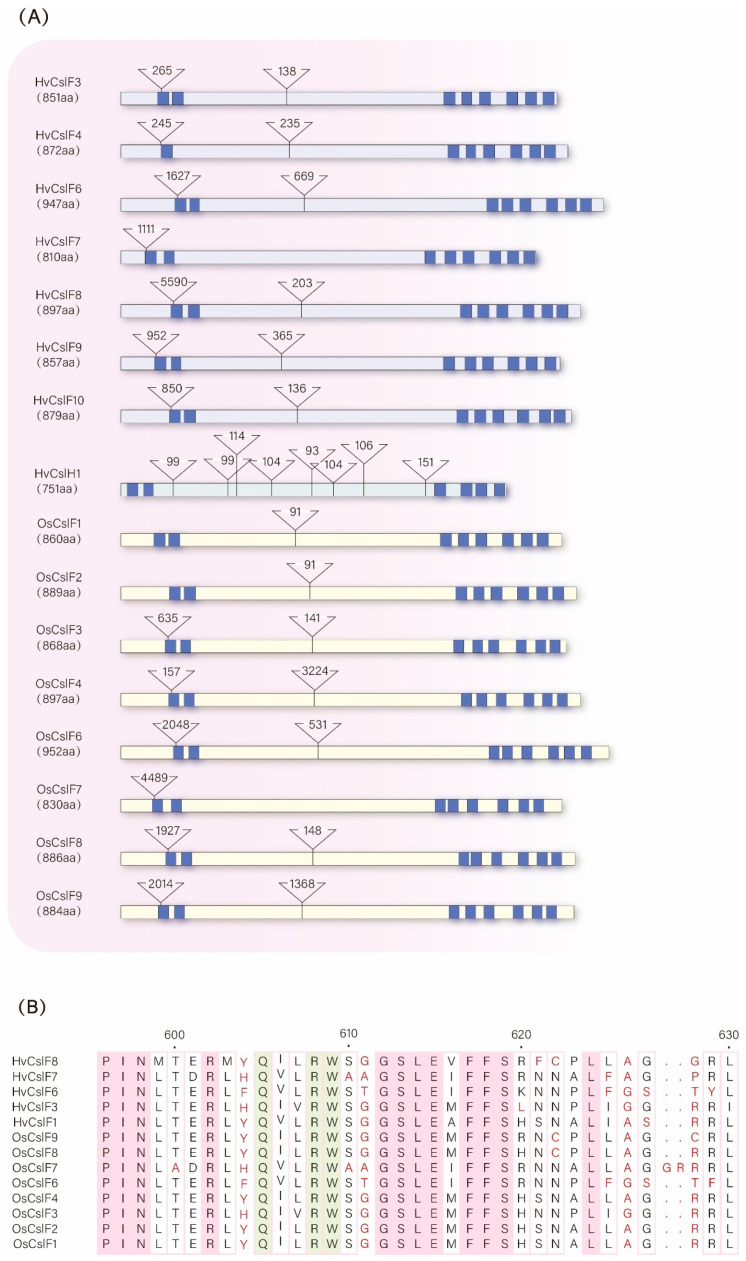
(**A**) Gene structures of *HvCslH1* [101] (green), *HvCslF* family members [101] (purple), and *OsCslF* family members [101] (yellow). The small dark blue boxes in A indicate the positions of sequences coding for transmembrane helices; triangles mark the positions of introns that do not participate in translation, and numbers in the triangles represent the size of introns (nt). (**B**) Multiple sequence comparison plots of *HvCslF* family members and *OsCslF* family members: green boxes mark the positions of the Q××RW motifs.

As shown in Figure 4A, the structural specificity of the *HvCslF* family is linked to its tissue-specific functions. Members of the barley *HvCslF* family (*HvCslF3/4/6/7/8/9/10*) differ significantly in gene length (810–947 aa) but all contain eight transmembrane helices (TMHs), with highly conserved positions and numbers of TMH-coding regions. This directly supports the conclusion in Section 3.2.1 that “*CslF* forms a transmembrane channel via eight TMHs to regulate the insertion frequency of β-1,3 linkages in MLG chains”. Notably, *HvCslF6* (947 aa) has the longest total intron length; as confirmed in Section 2.1.2, *HvCslF6* is the key gene for MLG synthesis in barley endosperm, and its extended introns may enhance post-transcriptional regulatory flexibility to support high expression during critical seed development stages.

*HvCslH1* shows structural complementarity with the *HvCslF* family. Barley *HvCslH1* (751 aa) has the same number of TMH-coding regions as *HvCslF*, but with a shorter total gene length and fewer introns. This aligns with its function of “synergizing with *CslF* for MLG synthesis” (Section 2.1.2), and its streamlined structure may enable rapid responses to *HvCslF* expression signals for functional complementarity.

There are structural differences in the *CslF* family between rice and barley. The TMH-coding region positions of rice *OsCslF* members (*OsCslF1/2/3/4/6/7/8/9*) are highly consistent with those of barley *HvCslF*, but some *OsCslF* members (e.g., *OsCslF6*, 952 aa) have significantly shorter introns than their barley homolog (*HvCslF6*). Combined with the conclusion in Section 2.2.1 that “the rice *CslF* family has simpler functions, mainly involved in MLG synthesis in leaves and roots”, this structural difference may lead to more efficient but less flexible transcriptional regulation of rice *CslF* genes, adapting to basic MLG synthesis in non-seed tissues rather than high MLG accumulation in barley seeds.

## 4. Regulatory Network of the (1,3;1,4)-β-Glucan Synthesis Gene Family

**Figure 5:**
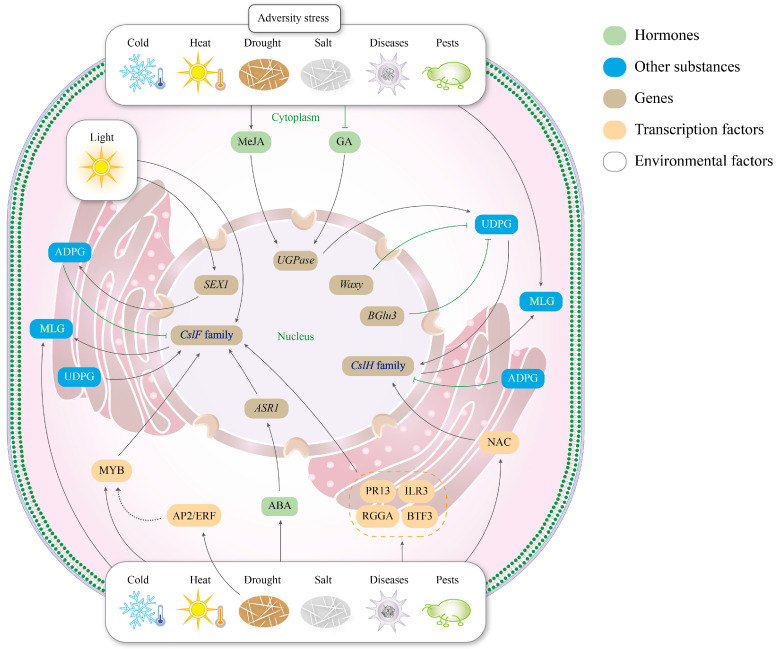
Regulation of β-glucan synthesis. The upper-right colors represent different regulatory factors; the black arrows represent promotion, and the green lines represent inhibition.

The functional implementation of β-glucan synthesis gene families depends not only on their own structures and mechanisms but also on coordinated regulation by external signals (e.g., hormones, environmental cues) and internal factors (e.g., transcription factors, key genes) (Figure 5). Below is a multi-dimensional analysis of the regulatory network of the (1,3;1,4)-β-glucan synthesis gene family.

### 4.1. Key Gene Regulation

The expression of the MLG synthesis gene family varies across crops and varieties. For example, the oat genome contains 76 Csl gene family members, among which the *CslF* and *CslH* subfamilies have adapted to the MLG synthesis needs of different tissues through gene duplication and subfunctionalization [50]. In contrast, the rice *Csl* gene family is smaller, with a relatively simple functional division [57]. In barley, the transcription of HvCslF genes exhibits significant varietal differences during endosperm development [15].

Genes involved in the MLG synthesis pathway also indirectly regulate the expression of MLG synthesis gene families. Mutations in the Waxy gene lead to the accumulation of UDPG-Glc, which in turn increases the MLG content [52]. *HvBGlu3* negatively regulates the β-glucan content in barley. As a *GH1* family β-glucosidase, *HvBGlu3* limits β-glucan accumulation in barley grains by hydrolyzing β-glucan or competing for sugar metabolism substrates [102]. Deletion of the SEX1 gene in barley increases the MLG content, which is associated with impaired ADP-glucose-to-starch conversion [99]. The main function of UDP-glucose pyrophosphorylases (UGPases) is to catalyze the formation of UDP-Glc. Overexpression of UGPases in transgenic Dendrobium ferrugineum significantly increases the soluble polysaccharide content [103], and a similar mechanism may exist in Gramineae crops.

### 4.2. Transcription Factor Regulation

Transcription factors directly regulate the expression of β-glucan synthesis genes (e.g., *CslF*, *CslH*) by recognizing cis-acting elements in their promoters and serve as “molecular switches” for MLG synthesis.

A variety of transcription factors regulate MLG synthesis. Among the MYB family transcription factors, OsMYB58 directly regulates MLG synthesis in rice endosperm by recognizing and binding to cis-acting elements in the *CslF* gene promoter [104]. In OsMYB58 overexpression lines, the MLG content increased by 18–22%, the endosperm cell wall thickness increased by 15%, and the rice drought tolerance was simultaneously enhanced [105]. HvMYB61 significantly upregulates the expression of *CslF6* by recognizing the TAACTG motif in the *CslF6* promoter [106].

In wheat, both *TaNAC29* and its homolog *TaNAC2D* target *TaCslH*. Mutation of either gene alone reduces the MLG content by only 8–10%, while a double mutation leads to a 25% reduction [107]. This indicates that family members ensure the stability of MLG synthesis through a redundant mechanism [108].

Among the members of the AP2/ERF transcription factor family, GhERF108 forms a complex with GhARF7-1/7-2, binds to the ERE+AuxRE composite element in the *GhMYBL1* promoter, and activates the downstream *CslD3* and *CslF6* genes [109]. In *Dendrobium catenatum*, CslG3 expression is induced by drought stress, and its promoter region contains binding motifs for AP2/ERF transcription factors—suggesting that the AP2/ERF family may regulate *Csl* gene expression via stress signaling pathways [105]. A similar mechanism may exist in the regulation of MLG synthesis gene families.

In addition, the binding of stress-responsive transcription factors—such as ILR3, BTF3, RGGA, and PR13—to CslF6 promotes MLG biosynthesis in barley [52]. Under iron-deficiency stress, ILR3 binds to the IDE1 motif in the *CslF6* promoter and upregulates CslF6 expression; after 48 h of iron deficiency, CslF6 transcription increases fourfold—linking iron metabolism to MLG synthesis [52]. As a core component of the RNA polymerase II complex, BTF3 maintains the constitutive expression of *CslF6*, and the basal MLG content in mutants decreases by 15% [52]. Induced by powdery mildew, RGGA/PR13 binds to the W-box in the *CslF6* promoter, promoting MLG deposition in stomatal guard cells. The MLG content in disease-resistant varieties is 32% higher than that in susceptible varieties [52]. ASR1 (abscisic acid stress ripening 1), a stress-responsive transcription factor, promotes β-glucan synthesis by positively regulating the expression of *CslF6* and increasing the concentration of UDPG substrates. It is involved in integrating hormonal signals such as ABA and environmental signals to regulate β-glucan metabolism [52].

### 4.3. Hormone Regulation

Plant hormones interfere with MLG synthesis through two pathways: “directly regulating substrate synthesis” or “indirectly activating transcription factors”. The core regulatory hormones are ABA (abscisic acid), GA (gibberellin), and MeJA (methyl jasmonate).

ABA-induced ASR1 enhances β-glucan synthesis by up-regulating the expression of *CsLF6* [52]. Gibberellin (GA) and methyl jasmonate (MeJA) directly promote UDPG synthesis by inducing the expression of *UGPase* genes, which promotes the expression of the MLG synthesis gene family [110]. This mechanism demonstrates how plants regulate MLG synthesis through hormonal signaling to maintain their growth and survival.

Combined with the transcription factor regulation discussed above, the ABA-MYB pathway exhibits a synergistic effect. ABA treatment can promote the phosphorylation modification of OsMYB58; the phosphorylated OsMYB58 shows a twofold increase in binding affinity to the OsCslF6 promoter, forming a dual activation pathway of “ABA→ASR1/OsMYB58→*CslF6*” to enhance the stress resistance response of MLG synthesis [52,104]. Additionally, there is a feedback balance between MeJA and AP2/ERF. While GhERF108 induced by MeJA activates *CslF6*, it can also bind to the promoters of key MeJA synthesis genes (e.g., LOX3) and inhibit their expression, thereby preventing abnormal cell wall rigidity caused by excessive MLG accumulation [109].

### 4.4. Environmental Conditioning

The expression of the MLG synthesis gene family is influenced by environmental factors, including light, temperature, moisture, salt stress, and pests and diseases, which also impact the expression of the β-glucan synthesis gene family.

Light exposure is one of the environmental factors influencing MLG synthesis, typically exerting its effect through the regulation of gene expression. For instance, under high light intensity, the expression level of the *AsCslF6* gene increases significantly [107], and light may also influence β-D-glucan synthesis by regulating the expression of the *SEX1* gene [111]. However, the precise mechanism by which light regulates the β-glucan synthesis pathway remains incompletely elucidated.

Temperature also has profound effects on MLG synthesis. For example, it has been shown that low-temperature stress tolerance in banana is associated with *MaCslA4/12*, *MaCslD4*, and *MaCslE2* [45]; the MLG levels were higher in plants grown under hot and dry conditions compared to cool and humid conditions [112]. Nevertheless, although Morgan has demonstrated that the MLG levels are higher under hot and dry conditions [112,113], Savin found that brief and very severe high-temperature stress may reduce the level of MLG [114].

Moisture also influences MLG synthesis. Viewpoints vary considerably among previous studies on the effects of moisture in the process of MLG synthesis. It has been found that MLG accumulation in oats increases in areas with low rainfall and severe water stress [115]. The MLG level in barley will reduce when it is grows with irrigation [116]; under drought conditions, the MLG content will reduce and stomata will close to limit transpiration [117]; and the MLG level will be higher under humid conditions [118]. MacNicol found that the timing of high-temperature or drought stress matters a lot and drought stress that occurs late in the grain-filling period has no effect on the MLG content [119].

Drought stress exhibits bidirectional effects. Some studies have shown that MLG accumulation in oats increases under conditions of low rainfall and severe drought [119], while the MLG content in barley decreases after irrigation [116]; however, other studies have found that drought leads to a reduction in the barley MLG content, with stomatal closure coinciding with MLG reduction [117]. Its MLG content is barely higher in a humid environment [118].

The reason for this contradiction may lie in differences in the stress timing, firstly. MacNicol pointed out that drought during the late grain-filling stage does not affect MLG, whereas drought during the seedling stage significantly alters its content [120], indicating that the crop developmental stage is a key variable. It also lies in cultivar specificity. The number of drought-responsive elements (e.g., abscisic acid-responsive elements [ABRE]) in the CslF6 promoter varies among different barley cultivars, which may lead to differences at the transcriptional level [52].

High-temperature stress exhibits complexity. Morgan found that the crop MLG content is higher under hot and dry conditions than under cool and humid conditions [113,114], but Savin observed that “short-term extreme high temperatures” reduce MLG levels [114]. This contradiction suggests that the “intensity and duration” of high temperatures are critical thresholds for regulating MLG—moderate high temperatures may promote substrate synthesis by inducing the expression of UGPase [110], while extreme high temperatures may disrupt the structure of the “switch motif” in *CslF6* [121], resulting in the loss of catalytic activity.

Salt stress usually affects the expression of genes related to MLG synthesis indirectly by regulating the overall plant metabolic network. For example, nitric oxide (NO) significantly promotes wheat fructan anabolism and regulates the expression of *SS* and *SBE* genes under low-temperature stress [122]. Although this study focused on fructans, the mechanism may apply to β-glucans because salt stress affects the synthesis of cell wall components through similar signaling pathways (e.g., Ca^2+^ fluctuations and ROS regulation) [123]. In addition, *CSL* gene expression is up-regulated and the MLG content increases in hydroponic sorghum under nitrogen deficiency [120].

Pests and diseases may induce MLG synthesis in crops, further supporting plant resistance to these threats. Pathogen infestation may induce the expression of endogenous β-glucan synthesis genes in plants; for instance, expression of the *HvCslF6* gene in barley correlates with disease resistance [124]. After brown planthopper (BPH, *Nilaparvata lugens*) attack, there is overexpression of *OsCslF6* encoding a glucan synthase that catalyzes the biosynthesis of mixed-bonded β-1,3;1,4-d-glucan (MLG), while the MLG levels are significantly increased in rice, significantly enhancing BPH resistance in vascular bundles [125]. Barley with high MLG content is resistant to the cereal cyst nematode [126]. Environmental stress affects β-glucan synthesis through multilevel regulatory mechanisms, enhancing crop survival in adverse conditions.

## 5. Summary and Outlook

β-glucan is a key functional polysaccharide in crop cell walls, whose synthesis relies on three major gene superfamilies: *Ces*, *CalS*, and *Csl*. Among these, *CslF/H/J* (unique to gramineous plants) are the core drivers of (1,3;1,4)-β-glucan (MLG) synthesis. These genes exhibit functional specialization and are regulated by multiple dimensions, including transcription factors, hormones, and environmental cues, providing molecular targets for crop improvement.

In the future, breakthroughs should be prioritized in four research directions. First, for *Csl* subfamilies with unknown functions (e.g., *CslE*, *CslG*, *CslM*, *CslB*), clarify whether they are involved in β-glucan or other cell wall polysaccharide synthesis by combining yeast or tobacco heterologous expression systems (e.g., the method used to verify *CslH* function [47]). Second, resolve the controversy over the subcellular localization of MLG synthesis: observe the dynamic colocalization of *Csl* proteins (e.g., *CslF6*) and MLG using super-resolution microscopy to resolve the contradiction between the “Golgi initiation” (in vitro synthesis experiments on maize coleoptile [28], localization observation of barley *CslF6* [77]) and “plasma membrane assembly” (MLG antibody labeling in gramineous plants [72]) hypotheses. Third, explore the regulatory mechanisms of environmental signals: analyze the regulatory pathways of *Csl* genes in response to the photoperiod (e.g., regulation by *SEX1* [99]) and drought [124,125] through integrated “environmental stress–transcriptome–metabolome” analysis (referring to the RNA-seq method used in oat seed development [73]), and establish an “environment–gene–MLG content” association model.

In addition, develop high-precision tools to dissect *CslF/H/J* functional redundancy [56,106], and leverage synthetic biology platforms for microbial β-glucan production while optimizing heterologous expression systems (e.g., the Golgi expression system for barley *CslF6* [77]) to boost in vitro MLG synthesis and industrial production as a natural additive [10]. In subsequent work, combine SNP markers (e.g., barley 7H’s B1_1033963 [127,128]) with genomic selection for high β-glucan crops adapted to marginal lands; use CRISPR to target *CslF6* and *HvCslH1* in breeding, or inhibit β-glucan hydrolases (e.g., *HvBGlu3* [102]) for precise MLG regulation in grains. These efforts will advance β-glucan applications in crop breeding, pharmaceuticals, and the food industries, aiding sustainable agriculture and health sector development.

## Figures and Tables

**Table 1:** Main functions and representative species of selected Csl families.

Subclass	Core Function	Representative Species	Tissues	Key References
CslA	It catalyzes the synthesis of hemicelluloses such as xyloglucan and mannan. Arabidopsis CslA7 is an essential protein for embryonic development (mediating glucomannan synthesis to support the embryonic cell wall).	*A. thaliana*	Embryo (CslA7), root (CslA3)	[38,39,40,41]
		*Dendrobium catenatum*	Stem (regulating polysaccharide accumulation)	
CslC	It is centrally involved in the synthesis of the xyloglucan main chain and regulates ethylene-mediated root growth inhibition in rice (balancing root elongation under ethylene stress).	*A. thaliana*	Leaves, roots (xyloglucan synthesis)	[32,40,41,42]
		*Hordeum vulgare*	Endosperm (formation of cell wall matrix)	
		*Oryza sativa*	Root (ethylene-responsive growth)	
CslD	Dual functions: synthesizing apical cellulose (supporting root hair/pollen tube tip growth) and mannan; banana MaCslD4 enhances cold resistance	*Linum usitatissimum*	Shoot apex (fiber development)	[43,44,45]
		*Prunus persica*	Fruit (mannan accumulation)	
		*Musa acuminata*	Leaf (cold resistance response)	
CslF	HvCslF6 in barley, a major synthase of the grass-specific (1,3;1,4)-β-glucan (MLG), dominates MLG synthesis in seeds, while HvCslF9 is a leaf pathogen-responsive protein.	*Avena sativa*	Stem, immature seeds (MLG accumulation)	[37,46]
		*Hordeum vulgare*	Endosperm (HvCslF6), leaf (HvCslF9)	
		*Oryza sativa*	Leaves, roots (OsCslF6 mediates MLG synthesis)	
CslH	Synergistically synthesizes MLG with CslF; double knockout of barley HvCslH1 and HvCslF6 leads to severe phenotypic defects (growth retardation, fragile cell walls)	*Hordeum vulgare*	Endosperm, leaf (HvCslH1 is involved in MLG synthesis)	[37,46,47]
		*Nelumbo nucifera*	Petiole (cell wall strengthening under salt stress)	
CslJ	Participates in MLG synthesis; in oats, co-expression with CslF promotes MLG accumulation, and there is convergent evolution with dicotyledon CslM	*Cornus florid*	Ye (MLG synthesis)	[37,46,48,49,50]
		*Avena sativa*	Stems, immature seeds (acting in synergy with CslF)	
		*Vitis vinifera*	Mature fruits (cell wall remodeling)	

**Table 2:** Comparison of β-glucan synthesis-related gene families.

Type	Species	Genome Size (Approximately)	Number of Members			Core Functions
			*CesA*	*CalS*	*Csl*	
Dicotyledon	*A. thaliana*	125 Mb	10 [61]	12 [62]	A/B/C/D/E/G (30 in total) [32,62]	*CesA*: synthesis of primary walls (*CesA*1/3/6) and secondary walls (*CesA*4/7/8); *CalS*: regulation of callose in pollen tubes and plasmodesmata [28,63]; *Csl*: synthesis of xyloglucan and mannan [32,63]
	*Gossypium hirsutum*	2.5 Gb	38 [64]	27 [65]	Only the existence of CslD (23 in total) has been clarified [66]	*CesA*: synthesis of fiber secondary walls; *Csl*: synthesis of pectin and hemicellulose; *GhCalS* responds to salt/heat stress [60,67,68]
	*Solanum* *lycopersicum*	900 Mb	16 [55,69]	Not clear	A/B/D/E/G (22 in total) [54]	*CesA*: cell wall formation during fruit development; *CslD*: cell wall formation during fruit development [54,70]
	*Medicago sativa*	800 Mb	10 [59]	12 [71]	Not clear (about 30 in total) [59]	*CesA*: synthesis of nodule cell walls; *Csl*: regulation of cell wall polysaccharides adapting to drought [59]
Monocotyledon	*Oryza sativa*	430 Mb	12	10	A/C/D/E/F/H (33 in total) [57]	*CesA*: stem fiber development; *CalS*: OsCalS5 regulation of anther callose [72]; *CslF*: MLG synthesis [73]
	*Zea mays*	2.3 Gb	12 [74]	10 [75]	A/C/D/E/F/H (56 in total) [76]	*CesA*: endosperm cell wall synthesis; *CslF*: MLG synthesis; *ZmCalS:* highly expressed in elongating roots/stems [77]
	*Hordeum* *vulgare*	5.1 Gb	9	7 [30]	At least A/C/D/E/F/H/J (30–40 in total)	*CesA*: endosperm cell wall synthesis; *CalS*: pollen development; *CslF/H/J*: MLG synthesis [51,56]
	*Avena sativa*	4.1 Gb	21 [78]	10 [78]	A/C/D/E/F/H/J (76 in total) [50]	*CslF/J*: stem, immature seed MLG synthesis; *CslH*: cell wall reinforcement under salt stress [50]

## Data Availability

No new data were created or analyzed in this study. Data sharing is not applicable to this article.
